# Excess Maternal Salt Intake Produces Sex-Specific Hypertension in Offspring: Putative Roles for Kidney and Gastrointestinal Sodium Handling

**DOI:** 10.1371/journal.pone.0072682

**Published:** 2013-08-22

**Authors:** Clint Gray, Emad A. Al-Dujaili, Alexander J. Sparrow, Sheila M. Gardiner, Jim Craigon, Simon J.M. Welham, David S. Gardner

**Affiliations:** 1 School of Veterinary Medicine and Science, University of Nottingham, Nottingham, United Kingdom; 2 Queen Margaret University of Edinburgh, Edinburgh, United Kingdom; 3 School of Biomedical Sciences, University of Nottingham, Nottingham, United Kingdom; 4 School of Biosciences, University of Nottingham, Nottingham, United Kingdom; Universidade Federal do Rio de Janeiro, Brazil

## Abstract

Hypertension is common and contributes, via cardiovascular disease, towards a large proportion of adult deaths in the Western World. High salt intake leads to high blood pressure, even when occurring prior to birth – a mechanism purported to reside in altered kidney development and later function. Using a combination of *in vitro* and *in vivo* approaches we tested whether increased maternal salt intake influences fetal kidney development to render the adult individual more susceptible to salt retention and hypertension. We found that salt-loaded pregnant rat dams were hypernatraemic at day 20 gestation (147±5 *vs.* 128±5 mmoles/L). Increased extracellular salt impeded murine kidney development *in vitro*, but had little effect *in vivo*. Kidneys of the adult offspring had few structural or functional abnormalities, but male and female offspring were hypernatraemic (166±4 *vs.* 149±2 mmoles/L), with a marked increase in plasma corticosterone (e.g. male offspring; 11.9 [9.3–14.8] *vs.* 2.8 [2.0–8.3] nmol/L median [IQR]). Furthermore, adult male, but not female, offspring had higher mean arterial blood pressure (effect size, +16 [9–21] mm Hg; mean [95% C.I.]. With no clear indication that the kidneys of salt-exposed offspring retained more sodium *per se*, we conducted a preliminary investigation of their gastrointestinal electrolyte handling and found increased expression of proximal colon solute carrier family 9 (sodium/hydrogen exchanger), member 3 (SLC9A3) together with altered faecal characteristics and electrolyte handling, relative to control offspring. On the basis of these data we suggest that excess salt exposure, via maternal diet, at a vulnerable period of brain and gut development in the rat neonate lays the foundation for sustained increases in blood pressure later in life. Hence, our evidence further supports the argument that excess dietary salt should be avoided *per se*, particularly in the range of foods consumed by physiologically immature young.

## Introduction

Between a quarter and a third of the world's adult population suffer from hypertension and as a result are significantly predisposed towards diseases of the cardiovascular system (CVD) including stroke, coronary and/or ischemic heart disease [1]. The prevalence of hypertension has changed little over the last three decades [2] and due to the increased morbidity and mortality associated with CVD, hypertension was estimated to cost the US economy alone $76.6 billion in 2010 [3].

The regulation of blood pressure is a classic example of a complex, multi-factorial and polygenic trait; hence, the aetiology of essential hypertension – a sustained above-average increase in blood pressure with no known cause – has occupied the hypertension field for many decades [4]. Whilst there is likely a genetic component [5] the majority of risk is conferred by environmental or ‘lifestyle’ factors such as diet. Of the multiple dietary factors that may influence blood pressure, the best available evidence indicates consumption of excess salt as a major contributor toward global hypertension [6]. Indeed, it has been proposed that a reduction of salt intake by 3 g/day would reduce the incidence of stroke by 13% and of ischemic heart disease by 10%, saving between 194–392,000 quality-adjusted life-years and $10–24 billion in health care costs annually [7]. Interventions and preventative medicine focussing upon reducing salt intake and its contribution to global non-communicable disease (particularly hypertension) are of the utmost importance [8].

Whilst increased salt consumption has demonstrable, direct pressor effects in adult individuals [9], [10], a proportion of the risk of an individual developing hypertension may be conferred by their developmental environment; that is, the immediate nutritional environment of the developing fetus-neonate-adolescent. For example, excess maternal intake of salt renders offspring with higher than average blood pressure as adults, despite no direct, excess intake of salt themselves [11]–[14]. Guyton first proposed the ‘pressure-natriuresis’ hypothesis as the main long-term mechanism for control of blood pressure [15]. One implication of this hypothesis was that a latent inability of the kidneys to effectively excrete salt over a wide range of intake (perhaps due to a ‘programmed’ deficit in function) drives an increase in arterial pressure, resulting in a higher pressor set-point (or ‘hypertension’) over which salt-balance is achieved. Whilst, animal models of diet-induced hypertension have generally supported a role for blunting of the renal pressure-natriuresis curve [16], recent data in humans has questioned its validity in the long-term (i.e. weeks-years) [17].

Nevertheless, the ability to excrete salt and participate in optimal fluid homeostasis is dependent upon the anatomical integrity and physiological capacity of the kidneys. Maternal malnutrition may impact upon both of these aspects of kidney function in the offspring, although a programmed deficit in nephron number has received most attention (for review see [18]). However, whilst such a phenotype may in the long-term contribute toward, or associate with, a tendency for increased blood pressure [19] the inherent physiological redundancy in glomerular number and volume (a 50–75% reduction is required for clinical signs [e.g. azotaemia] to become apparent) suggests that multiple functional deficits must also parallel any anatomical compromise. A number of animal studies have shown maternal diet to lead to disturbed renal electrolyte handling in the offspring [18], although a consistent renal phenotype has failed to emerge, perhaps reflecting the low signal-to-noise ratio in nutritional studies of the developmental programming paradigm. In contrast, maternal intake of excess salt has been shown on a number of occasions to increase blood pressure in the adult male and/or female offspring [12]–[14], although a lack of effect has also been observed [11]. The mechanism for any pressor effect of excess maternal dietary intake of salt on the next generation has also remained elusive.

Hence, in this study we chose to use the paradigm of maternal salt-loading to examine the programming of renal function and blood pressure in the adult (12 week old) male and female offspring. We hypothesised that maternal salt-loading would lead to hypernatraemia in both dam and fetus which blunts development of the fetal kidney and compromises adult renal function; an effect characterised by renal sodium retention, hypernatraemia and hypertension in both male and female offspring. We used a combination of *in vitro* and *in vivo* approaches (using multiple cohorts) to examine the effect of high extracellular salt on kidney development and function. After characterisation of our phenotype partially supported the null hypothesis (i.e. that under conditions of normal dietary salt, increased renal sodium retention could not explain hypernatraemia in both male and female offspring of salt-loaded dams and hypertension in male offspring only) a further cohort was established in which we repeated our analyses of renal function in the offspring (at 8 weeks of age) but also conducted a preliminary analysis of gastrointestinal electrolyte handling and faecal characteristics in the male and female offspring at this time.

## Materials and Methods

All procedures involving animals were carried out under license and in accordance with the Home Office Animals (Scientific Procedures) Act 1986 and were approved by the local ethical review committee of the University of Nottingham. The study was designed to test the hypothesis that maternal intake of excess salt impedes normal development of the fetal kidney leading to poorer control of salt balance later in life, an effect mediated at the level of the kidney.

### Experimental procedures-dams

59 Sprague Dawley female rats (190–200 g; 8–10 weeks of age; Harlan, UK) were housed in a temperature (20–22°C) and humidity (55–65%) controlled environment and subjected to a 12 hour light/dark cycle (0700–1900 h). Dams were fed *ad libitum* standard laboratory chow (AIN-93G, Harlan) for 1 week prior to being randomly assigned to 1) Control diet (CD; 0.26% NaCl, n = 33) fed purified standard chow (TD.08164; Teklad Harlan, Maddison. WI.) and tap water or 2) Salt diet (SD; 4% NaCl, n = 26) fed purified standard chow with 4% NaCl added (TD.08162 Teklad Harlan, Maddison WI.) and tap water. Rats were habituated to the diets for 4 weeks and remained on the diets through mating, conception (plugging designated as d0), gestation and lactation (offspring weaned at 3 weeks of age). Weight gain and other descriptive parameters in dams were not influenced by diet (data not shown). Proportions of dams were euthanized (rising concentration of CO_2_ with cervical dislocation) at different stages of gestation (4 days [CD, n = 10; SD, n = 10] and 20 days [CD, n = 10; SD, n = 6]; term, 21±1 days) for blood collection (into Li-heparin tubes) and plasma. At day 20 gestation, maternal and fetal organs were recovered and either snap frozen in LN_2_ (stored at −80°C) or fixed (4% PFA, 24 h at 4°C) and plasma obtained (stored at −20°C). Remaining dams (CD, n = 13; SD, n = 10) proceeded to term with litters standardized to eight pups at birth (4 female, 4 male). At weaning, dams were euthanized and the remaining pups group housed according to sex and fed standard chow diet thereafter, unless otherwise indicated. Due to occasional experimental difficulties not all measurements were available for all variables in dams and the appropriate experimental *n* is indicated in individual Figures and Tables.

### Experimental procedures-offspring

After weaning and between 8–12 weeks of age, two siblings from each litter (one male, one female) were entered into one of four protocols:

1. **Baseline renal function at 8 and 12 weeks of age.** Baseline renal function was established in two cohorts of offspring at 8 and 12 weeks of age (control diet, male [n = 6] female [n = 5]; 4% NaCl, male [n = 5] female [n = 5]) by 24 h urine collection in a metabolic crate (after 24 h acclimatisation to the environment) with a paired blood sample collected at 24 h.
2. **Salt-stimulated renal function at 12 weeks of age.** In a separate cohort, salt-stimulated renal function was established in 12 week old offspring (control diet, male [n = 6] female [n = 5]; 4% NaCl diet, male [n = 5] female [n = 5]). In brief, renal function was assessed as described above but after rats were fed salt-diet for 4-days (including 24 h acclimatisation to the met crate).
3. **Blood pressure assessment by telemetry.** A proportion of offspring (control diet, male [n = 6] female [n = 5]; 4% NaCl, male [n = 5] female [n = 5]) were surgically implanted with a radiotelemetric probe at 9 weeks of age, as previously described [20]. In brief, the rats were fully anaesthetised (fentanyl citrate; Sublimaze, Janssen-Cilag and medetomidine hydrochloride; Domitor, Pfizer, UK; 300 ug.kg^−1^ of each i.p.), for probe implantation (TA11PA-C40; DSI, St-Paul, MN USA) as described previously [20]. Anaesthesia was reversed (Antisedan, Pfizer UK; 1 mg kg^−1^) and analgesia administered (buprenorphine; Buprecare, Animalcare UK; 0.02 mg kg^−1^ s.c.) together with a long-acting antibiotic (Amoxycare LA; 0.05 ml *i.m.*). After 5–7 days, blood pressure was measured continuously for a 48 h period at a sampling rate of 2×15 sec periods per min (Dataquest A.R.T GOLD v4.02; DSI, USA).
4. **Tissue collection at 8 and 12 weeks of age.** Any remaining offspring from each cohort (Control diet, male [n = 6] female [n = 5]; 4% NaCl, male [n = 5] female [n = 5] at each timepoint) were euthanised at 8 or 12 weeks of age without exposure to any experimental procedure for collection of control (unstimulated) plasma and organs. At 8 weeks of age, faecal matter (the first fully formed stool in the colon) and gastrointestinal tissue (proximal and distal colon) were specifically collected for fixation (4% PFA) or snap-frozen in LN_2_ and stored at −80°C for later analysis.

### Experimental measurements

All dams and/or offspring were euthanised between 09.00–11.00 hrs. Plasma or urinary osmolality was determined by freezing point depression (Osmomat 030, Gonotech, UK) with intra-assay variability being <1%. Tissue dry weights were determined by freeze-drying. Biofluid and faecal electrolytes (Na, K and Ca) were determined by inductively-coupled plasma mass spectrometry (ICP-MS; XSeries II, Thermo Fisher, Ltd) with intra-assay variability being <2%. Faecal matter was first acid-digested (6 ml concentrated HNO_3_) using a microwave (1400 W for 25 mins; Anton Paar Multimave 3000) followed by addition of 4 ml H_2_0. Physiological measurements are presented adjusted to body weight (kg BW) to allow informed assessment of between-sex differences (males being much larger than females). Urine flow rate (V_urine_) was measured in ml per 24 h and is presented as ml/min/kg BW. Creatinine (C_cr_), albumin (C_alb_) or osmolal (C_osm_) clearance (ml/min/kg BW) were calculated as (e.g. for creatinine; [Cr_urine_* V_urine kg BW_]/1440)/Cr_plasma_). Free water clearance (C_H2O_) was calculated as urine flow rate – C_Osm_.

### Hormone assays

Plasma and urinary corticosterone and aldosterone were measured by ELISA as described by [21], [22]. Plasma arginine vasopressin (AVP) was measured by ELISA following the kit instructions (arg^8^-vasopressin EIA; Enzo Life sciences, Exeter, UK). Plates were read at 450 nm (corticosterone, aldosterone) or 405 nm (vasopressin) on an ELISA MRX plate reader. Values were interpolated from a 4-parameter logistic curve and reported cross reactivity for vasopressin was <0.001% (oxytocins, enkephalins and other related peptides). The reported minimum detection limit for the hormones was: corticosterone, 6.6 pg/ml; aldosterone, 3.4 pg/ml; vasopressin, 3.39 pg/ml.

### Nephron numbers

Rat glomeruli were counted at day 20 gestation and at 8 weeks postnatal age as described previously [23]. In brief, whole kidneys were incubated in 1 mol/L hydrochloric acid for 30 minutes at 37°C, acid was replaced with 0.5 mL PBS and tissue homogenized. 20 µL homogenate was visualised on a slide with a ×10 objective and the total number of glomeruli counted. The procedure was carried out in triplicate for each sample and has a typical intra- and inter-assay variation of 10% and 11%, respectively.

### Organ explant culture

Female mice (outbred ICR strain) were time mated between 09.00 hrs and 13.00 hrs and at embryonic day 12 dams were euthanized and the fetal kidneys/lungs were removed and cultured on polyethylene terephthalate tissue culture plate inserts (Millipore Corporation, USA). Cultured kidneys were maintained in DMEM-F12 (Sigma, UK; 330 mosmoles/L) supplemented with insulin (10 mg/L), transferrin (5.5 mg/L), sodium selenite (5 µg/L), penicillin and streptomycin. Cultured lungs were maintained in BGjB medium Fitton-Jackson Modification (Invitrogen, Paisley, UK) containing 25 mg/dl ascorbic acid and 1% heat inactivated fetal bovine serum (Invitrogen, Paisley, UK). All organs were maintained at 37°C in 5% CO_2_. Sodium chloride, mannitol or urea was added to the media to achieve an increase in ECF osmolality of 0 mM, 25 mM, 50 mM, 100 mM, 200 mM; 25 mM NaCl being reflective of the differences in plasma osmolality observed *in vivo* after salt-loading, whereas the higher concentrations were used to illustrate the toxic effect of high-salt on organ development. Organs were imaged using light microscopy (Leica Microsystems, UK) and surface area determined with Image Pro Plus (Media Cybernetics Inc. USA).

### Western blotting and PCR

Western blotting was carried out as previously described [24]. Briefly, tissues were disrupted in 5 volumes of ice-cold homogenisation buffer (150 mM NaCl, 50 mM HEPES, 2.5 mM EDTA, 10% glycerol, 1% Triton, 1 mM Na_3_VO_4_, 10 mM NaF) containing a protease inhibitor cocktail (Roche Diagnostics, West Sussex, UK). Approximately 50 µg protein was probed with an antibody raised against 11β hydroxysteroid dehydrogenase type II (Abnova, CA, USA). Bands were visualised using ECL Advance reagent (GE healthcare, Amersham, UK). Standard PCR was used to visualise the presence or absence of SLC9A3 in proximal or distal colon (×30-cycles) according to the manufacturers protocol (Sigma-taq) using the primer sequence: forward, TATCTTCGCCTTCCTGCTGT; reverse, GCTCTGAGATGTTGGCCTTC. 18S was used as the internal control.

### Statistics

The study was designed and analysed as a 2 (salt, yes/no)×2 (sex, male/female) factorial ANOVA. Data and residual distributions were first checked and log_10_-transformed before analysis, as required. Data are presented as estimated marginal means from the model with ± standard error of the mean (SEM) or of the difference between means (s.e.d.) or 95% confidence interval, as appropriate to represent the error for each comparison. Where male and female siblings were included in the statistical model then the dam was added as a random effect (to account for reduced intra-litter variance) and data were analysed by a General Linear Mixed Model (GLMM; Genstat v14, VSNi, UK). For cardiovascular circadian analyses all continuously recorded cardiovascular data (e.g. 2880 datapoints per animal per day; 14,400–17,280 datapoints per group [n = 5–6 animals of each sex] were entered into a non-linear regression model fitting a Fourier-curve (Y = α+β*sin*(2π(X+ε)/w) to derive four parameters *α*, set-point; *β*, amplitude; *w*, wavelength and ε, offset, which were analysed by 2-way ANOVA.

## Results

### Dietary salt-loading leads to maternal hypernatraemia

In rats fed excess dietary salt for 4 weeks prior to conception and to day 4 gestation, plasma osmolality was significantly increased (296±2 *vs.* 278±2 mosmoles/kg H_2_O for SD *vs.* CD dams, respectively). Continued dietary salt-loading maintained this difference in maternal plasma osmolality, for example, when measured at day 20 gestation (Table 1) or at weaning (315±4 *vs.* 296±5 mosmoles/kg H_2_O for SD *vs.* CD dams, respectively). With no difference in plasma glucose, albumin or urea between diet groups (data not shown) the diet-induced difference in osmolality was likely due to increased extracellular fluid (ECF) sodium, an effect confirmed when measured at day 20 gestation (plasma [Na^+^] was 147±6 in SD *vs.* 121±6 mmoles/L in CD dams, mean ±S.E.M. For comparison, in our hands measured plasma sodium in non-pregnant rats (n = 5) is 143±8 mmoles/L. At day 20 gestation, salt-loaded pregnant rat dams had renal hypertrophy (5.32±0.10 *vs.* 4.18±0.13 mg/g for SD *vs.* CD dams, respectively; P<0.001) accompanied by polydipsia and polyuria with significantly increased free water clearance (Table 1). Thus, despite marked osmolar clearance and cation (particularly Na^+^) excretion (Table 1), plasma osmolality remained significantly elevated in salt-fed dams, due to hypernatraemia. We speculated that maternal hypernatraemia would significantly impact development of the fetal kidneys and tested this hypothesis using *in vitro* and *in vivo* systems.

**Table 1:** Maternal salt diet has a marked impact on renal function in the pregnant dam.

*Plasma and urinary biochemistry in pregnant dams at day 20 gestation*			
	Control	4% salt	*P*
**Food intake (g/kg BW/day)**	60.2±5.5	61.8±4.6	NS
**Water intake (ml/kg BW/day)**	74.6±19.8	151±18	0.003
**Urine volume (ml/kg BW/day)**	26.8±5.7	113.3±5.3	<0.001
**Plasma osmolality (mosmoles/kg H_2_O)**	275±4.7	294±4.6	0.006
**Urine osmolality (mosmoles/kg H_2_O)**	1453±71	1094±44	<0.001
**Na excretion (µmoles/h/kg BW)**	32±7.6	1743±161	<0.001
**K excretion (µmoles/h/kg BW)**	110±56	197±43	NS
**Creatinine clearance (ml/min/kg BW)**	2.21±0.20	2.60±0.18	NS
**Osmolar clearance (ml/min/kg BW)**	0.10±0.02	0.27±0.01	<0.001
**Free water clearance (ml/min/kg BW)**	28.4±6.0	113±5.0	<0.001

Food and water intake were measured daily, values represent the average intake at day 20. A 24 h urine collection with paired blood sample enabled analysis of renal function. Osmolarity, creatinine and electrolytes were measured by an osmometer (Osmomat 030, Gonotec), auto-analyser (RX-IMOLA, Randox) and ICP-MS (XSeries II, Thermo Fisher, Ltd), respectively. Data are means ±SEM for n = 8 dams per dietary group and were analysed by 1-way ANOVA for an effect of treatment (Genstat v14). Statistical significance was accepted at P<0.05. NS, not significant. BW, body weight.

### Elevated sodium chloride in culture media significantly impedes branching morphogenesis in the kidney, but not lung

**Figure 1:**
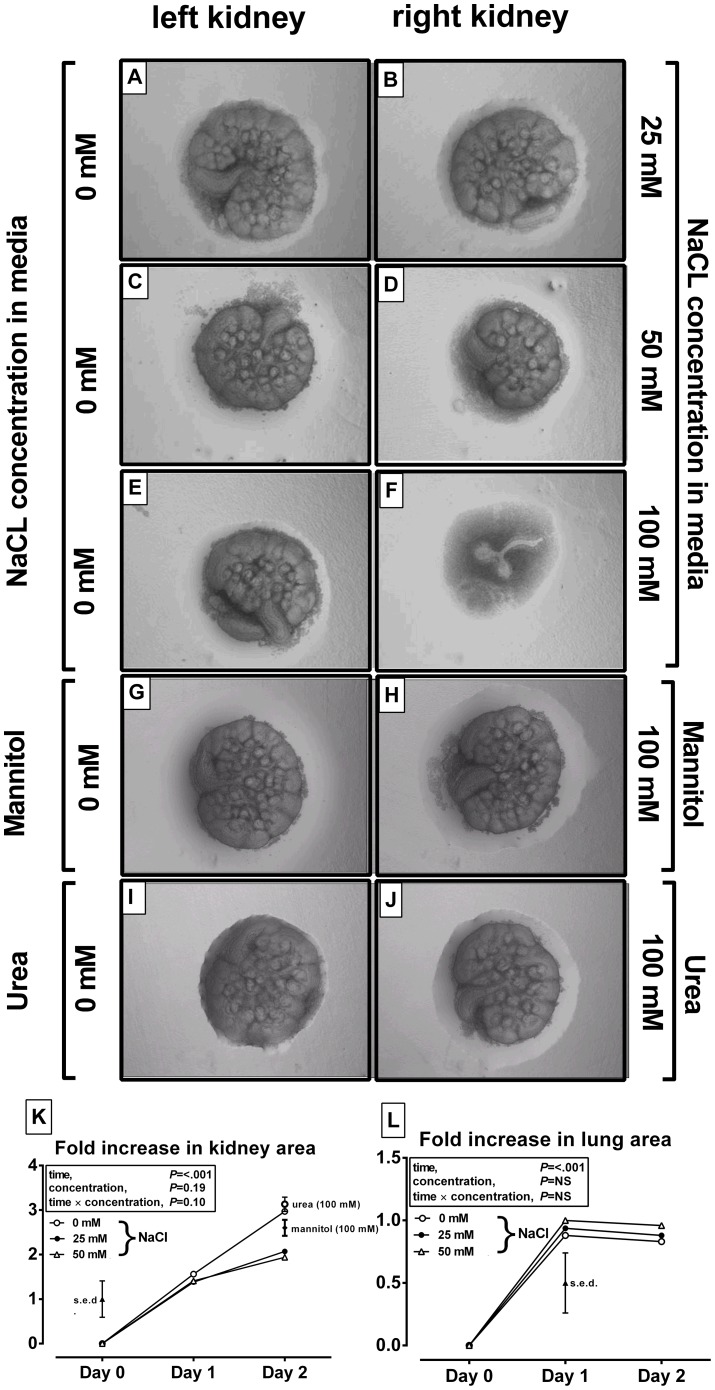
Increased extracellular salt blunts in vitro kidney but not lung development. **A–J**: representative images of paired (left and right) kidneys (n = 4–6 replicates for analysis) cultured for 2 days in media with varying osmolality, generated using NaCl, mannitol or urea, at concentrations indicated on *y*-axes. **K**: growth (fold-increase in normalised surface area) of cultured kidneys or lungs (**L**). Estimated marginal means for data are presented after analysis by repeated measures general linear models with treatment (NaCl, mannitol or urea) and concentration (0, 25, 50, 100 mM) or specific interactions as fixed effects and time as a repeated measure (Genstat v14). The overall standard error of the difference (s.e.d.) between means for the statistical comparison is presented.

Using an organ explant culture system, murine E12 fetal kidneys and lungs were grown in the presence or absence of varying concentrations of NaCl or alternative osmolytes known to cross (e.g. urea) or not cross (e.g. mannitol) plasma membranes. When grown in NaCl for two days, growth of murine fetal kidneys was reduced at 25 mM NaCl (Figure 1B) but markedly blunted at 50 mM (Figure 1D,K) and effectively arrested at 100 mM (Figure 1F). The osmotic pressure exerted by NaCl is double its molar concentration suggesting that at 12–25 mM NaCl, or a 25–50 mosmole/kg increase in NaCl in the culture media, is sufficient to reduce branching morphogenesis in the developing kidney (Figure 1K). In order to separate an osmotic from a direct effect of Na^+^ *per se*, we cultured organ explants in the presence of either mannitol or urea at 100^+^ mosmoles/kg. At 100 mosmoles/kg, and for both substances, there was no blunting of renal branching morphogenesis (Figure 1H,J). To determine, whether these effects were specific to the kidney, the *in vitro* experiment was replicated in fetal lung explants, another organ exhibiting branching morphogenesis. At higher NaCl concentrations in the media (e.g. 100 mosmoles NaCL) the culture media tended to impede *in vitro* lung growth (Figure S1J–L), but below this level (e.g. 25–50 mosmoles NaCl) branching morphogenesis of the lung was not obviously affected (Figures, S1D–I). Thus, elevated sodium chloride – within a physiological range - significantly blunts branching morphogenesis in the kidney, but not lung, and thus restricts their developmental potential. However, the extent to which hypernatraemia in ECF may impact kidney development *in vivo* is not known and was therefore tested in our nutritional model.

### Maternal hypernatraemia is not reflected in the the fetal environment and thus has little effect on in vivo fetal renal development

Fetal plasma osmolality was similar in both male and female fetuses, and was not influenced by maternal salt diet (301±1 *vs.* 298±1 mosmoles/kg H_2_O for SD *vs.* CD fetuses, respectively). In addition, glomerular number, a marker for the degree of branching renal morphogenesis at this time was not different between treatment groups (males, 1166±110 *vs.* 1066±95 glomeruli; females, 1121±94 *vs.* 966±156 glomeruli for SD *vs.* CD fetuses, respectively). Furthermore, fetal and placental (wet and dry) weights were also not different between treatment groups or sex (Table 2). In all groups, fetal body water content diminished at birth, relative to day 20, but this was unaffected by maternal salt intake (Table 2). Thus, *in vivo* at 0.95 gestation, the developing fetal kidney appears relatively spared from the effects of maternal hypernatraemia. However, in the altricial, polytocous rat the kidneys continue to develop until 1.33–1.47 gestation (postnatal day 7–10) and the maternal diets are fed throughout this time (to weaning at day 21). Hence, further potential effects of maternal salt diet on renal structure and function of the subsequent adult offspring were investigated.

**Table 2:** Maternal salt diet has little effect on feto-placental tissues in late gestation.

*Tissue composition of feto-placental unit ay day 20 and term (day 21)*							
		± Maternal salt			*P* value		
Males	Age	−ve	+ve	s.e.d.	Salt	Age	Salt*Age
**Fetal wet weight (g)**	**d20**	3.51	3.58	0.20	NS	<.001	NS
	**term**	5.49	5.67				
**Placental wet weight (mg)**	**d20**	527	547	36	NS	-	-
	**term**	-	-				
**% water in fetus**	**d20**	84.2	87.1	1.6	NS	0.02	NS
	**term**	81.8	82.9				
**% water in placenta**	**d20**	84.4	85.4	0.62	NS	NS	NS
	**term**	-	-				
**Females**							
**Fetal wet weight (g)**	**d20**	3.50	3.49	0.20	NS	<.001	NS
	**term**	5.54	5.49				
**Placental wet weight (mg)**	**d20**	493	527	36	NS	-	-
	**term**	-	-				
**% water in fetus**	**d20**	85.0	86.6	1.6	NS	0.02	NS
	**term**	83.2	83.0				
**% water in placenta**	**d20**	84.8	85.2	0.62	NS	NS	NS
	**term**	-	-				

Data are estimated marginal means plus the standard error of the differences between means (s.e.d.). At day 20, data are n = 10 dams per dietary group (Control, n = 4 male/female pups; 4% salt diet n = 7 male and n = 4 female pups). At term, data are n = 8 control and n = 5 SD dams (Control, n = 8/8 male/female pups; SD diet, n = 7/5 male/female pups). Data were analysed by mixed effects models with treatment (control *vs.* 4% salt), sex (male *vs.* female) and age (day 20 *vs.* term) or their interaction as fixed effects and dam as a random effect (Genstat v14). Log_10_ transformation was used before analysis, as required, to achieve normality in error distribution. NS, not significant. −ve, is control diet (CD). +ve, is 4% salt diet (SD).

### Maternal dietary salt-loading leads to hypernatraemia and hypertension in the adult offspring

**Figure 2:**
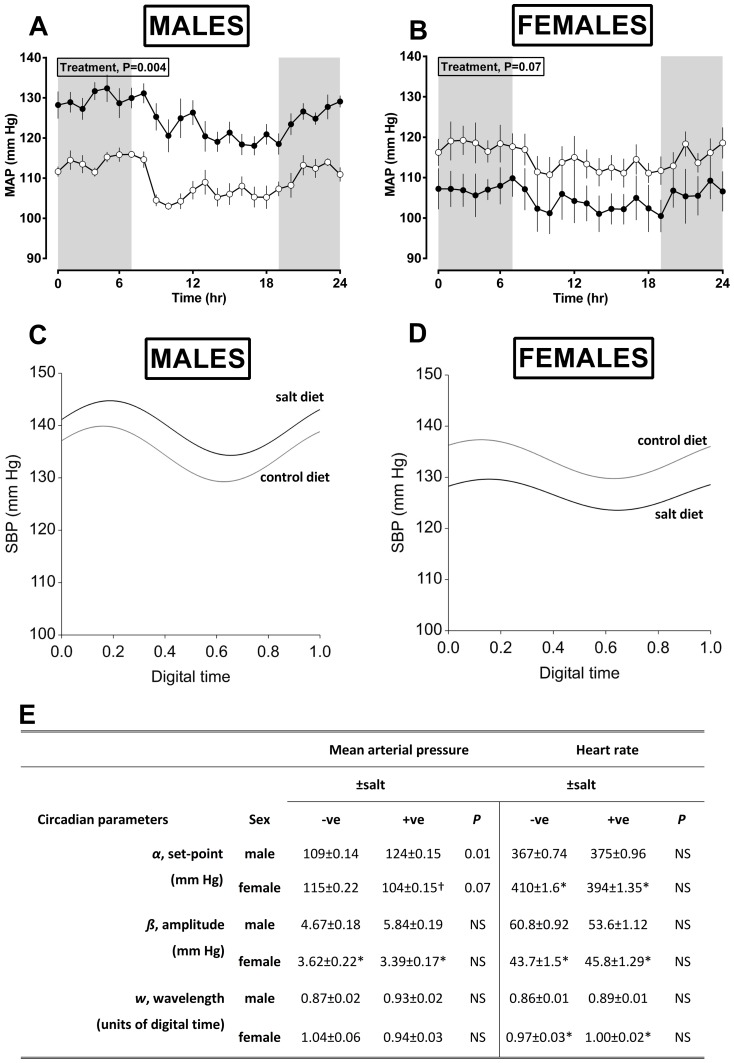
Salt-exposed offspring have increased blood pressure throughout the circadian cycle. For blood pressure, assessed by telemetry, data are from Control diet; n = 6 dams, n = 6/6 [male/female] offspring or high-salt (4% salt) diet; n = 6 dams, n = 5/5 [male/female] offspring. Values are either hourly means (A,B; derived from summary measures of 48 h recording) or the fitted model from non-linear regression [Fourier] analysis of all datapoints recorded over the 48 h period (C,D) and the respective output of circadian parameters from the model (E). Data are predicted means (±S.E.M.) and were analysed by mixed effect models with treatment (control *vs.* 4% salt) and sex (male *vs.* female) or their interaction as fixed and dam as a random effect (Genstat v14). NS, not significant.

Despite no overt exposure to excess dietary salt since weaning (a 9-week interval), the male and female prenatally salt-exposed offspring had significantly increased plasma sodium concentration (163±4 *vs.* 149±2 mmoles/L for SD *vs.* CD offspring, respectively) and thus osmolality (322±2 *vs.* 361±3 mosmoles/kg H_2_O for SD *vs.* CD offspring, respectively) with no influence of offspring sex being evident (hence, data for males and females combined). The adult male offspring of salt-fed dams had significantly higher blood pressure throughout the circadian cycle whilst female offspring tended to have chronically lower blood pressure relative to respective control offspring (treatment × sex effect; 25±6 mm Hg; Figure 2A–D). Derivation of circadian parameters by Fourier analysis of all measured datapoints confirmed a treatment × sex interaction on the set-point for mean arterial pressure and indicated females *per se* to have a reduced pressor and chronotrophic amplitude (Figure 2E). Given the role of the kidneys in salt-balance we investigated a potential renal mechanism for increased salt retention in prenatally salt-exposed offspring under baseline and salt-loaded conditions i.e. after 5 days feeding of a low (0.26%; standard chow) or high-salt (4%; TD.08162) diet.

### Hypernatraemia in maternally salt-exposed offspring is unlikely due to blunted renal excretion of Na

**Figure 3:**
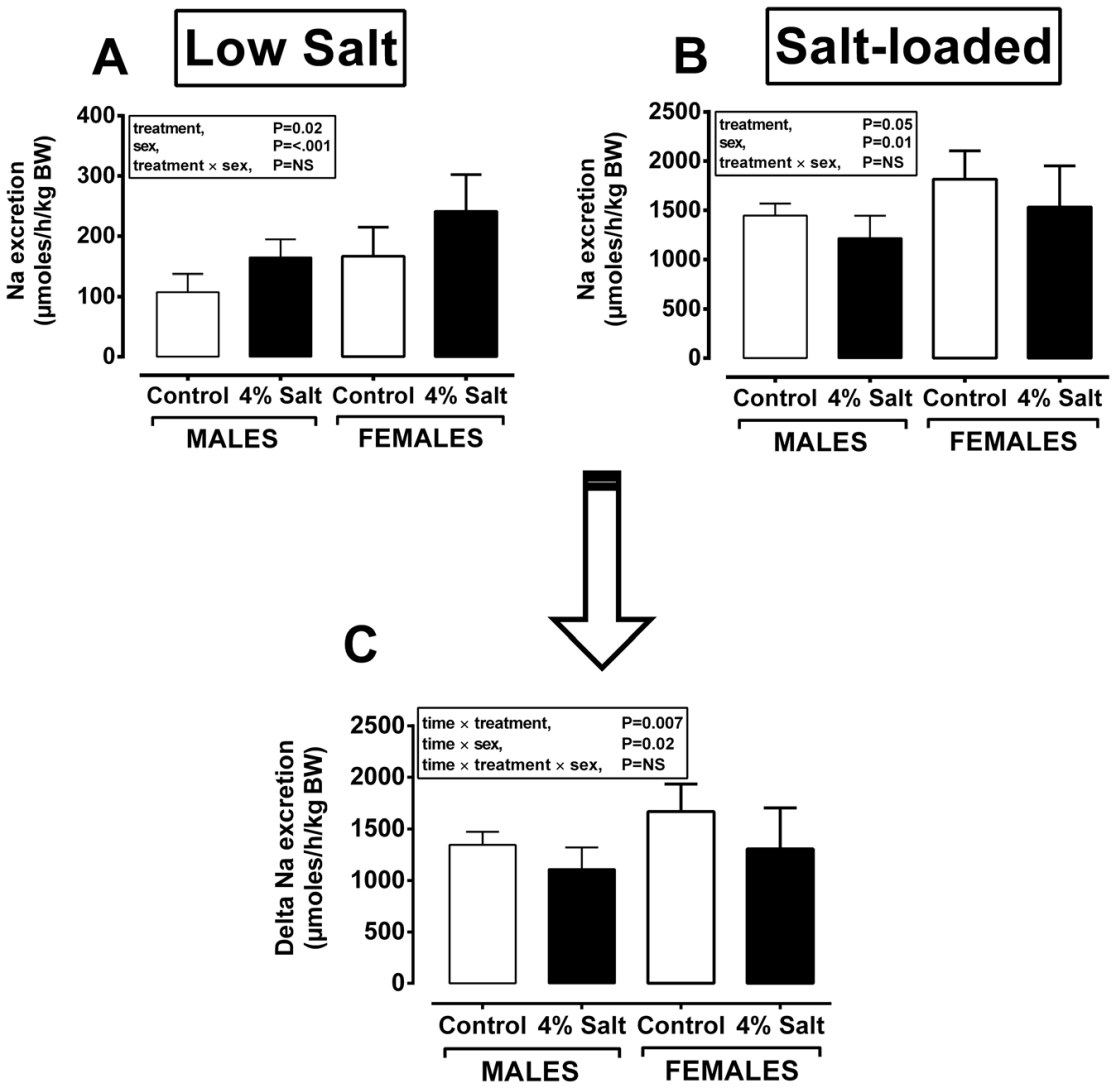
Salt-exposed offspring do not retain excess salt but have greater sodium excretion under low, but not high, salt-loading. Na^+^ excretion were measured in 12 week old male and female offspring from dams fed control diet (Control, n = 12 dams; n = 9–12 males/females) or 4% salt diet with water *ad libitum* (4% Salt, n = 10 dams; n = 7–10 males/females). Paired plasma and urine were collected after 24 h in a metabolic crate after 5-days feeding either a low (0.26%; standard chow or LOW-SALT) or high-salt (4%; TD.08162, SALT-LOADED) diet. Data are means (±95% CI) and were analysed by mixed effect models with treatment (control *vs.* 4% salt) and sex (male *vs.* female) or their interaction as fixed and dam as a random effect (Genstat v14). NS, not significant.

Food and thus dietary salt intake together with markers of baseline renal function in prenatally salt-exposed *vs.* control adult offspring were not different between treatment groups. On the low salt ‘chow’ diet, hypernatraemia in prenatally salt-exposed offspring appeared to be partially accommodated by increased renal Na^+^ excretion (Figure 3A), contributing toward greater total osmolal excretion in this group (males, 2644±275 *vs.* 2191±265; females, 3767±275 *vs.* 3466±260 mosmoles/h/kg BW for SD vs. CD, respectively; P*_treatment_* = 0.007). When adjusted to plasma osmolality (i.e. osmolar clearance) the treatment effect disappeared (Table 3 [8 weeks of age] and Table 4 [12 weeks of age]), suggesting normal renal function under baseline conditions and when consuming a low-salt ‘chow’ diet. Thus, in order to ascertain if the kidneys of prenatally salt-exposed offspring more efficiently retain dietary Na^+^ under salt-loaded conditions, we subjected the offspring to a high salt diet (i.e. equivalent to their parents diet) for 5 days and repeated the renal functional analyses. Dietary salt loading did not have a significant additional effect on plasma osmolality of offspring (356±7 *vs.* 322±7 mosmoles/kg H_2_O for SD *vs.* CD, respectively; P*_treatment_* = 0.005), but as expected, resulted in polydipsia and polyuria in all animals (Table 5) with Na^+^ excretion increasing approximately 10-fold; however, the calculated increment in Na^+^ excretion with salt-loading was less in prenatally salt exposed offspring (Figure 3C) suggesting a tendency toward greater salt-retention under conditions of salt-loading in these animals. Functional differences in the adult offspring of salt-exposed dams at this age (8–12 weeks) were not paralleled by any renal anatomical differences; for example, relative kidney weights (males, 6.84±0.13 *vs.* 6.76±0.12; females, 6.64±0.13 *vs.* 6.71±0.13 g/kg BW for SD vs. CD, respectively) were similar, renal histology did not indicate any glomerular or tubular injury/hypertrophy or adverse infiltration of inflammatory cells (Figure S2A,B) and the nephron complement was similar between treatment groups (Figure S2C). Furthermore, hypernatraemia in prenatally salt-exposed offspring was not accompanied by any change in plasma aldosterone (Figure S2D) and plasma vasopressin concentrations were below the limit of detection in all animals. In addition, we found no difference in the protein abundance of the glucocorticoid metabolising enzyme 11β-HSD2 in the kidneys of prenatally salt-exposed offspring (Figure S2G) and hence, no difference in their 24 h excretion of corticosterone (Figure S2E) or aldosterone (Figure S2F). Taken together, the data corroborate the lack of a significant renal phenotype mediating hypernatraemia in prenatally salt-exposed offspring. The other major site for reabsorption of sodium and thus water is the distal colon; therefore we conducted a preliminary study investigating gastrointestinal electrolyte handling in the adult (8 weeks of age) offspring of control and salt-exposed offspring.

**Table 3:** Offspring renal function is unaffected by maternal salt-exposure.

*Renal functional parameters at baseline in adult offspring at 8 weeks of age*							
		± Maternal salt			*P* value		
	sex	−ve	+ve	s.e.d.	Salt	Sex	Salt*Sex
**Urine output (ml/min/kg BW)**	**male**	20.0	30.1	5.3	0.07	<.001	NS
	**female**	33.9	43.3				
**Osmolal clearance (ml/min/kg BW)**	**male**	0.05	0.07	0.1	NS	0.002	NS
	**female**	0.11	0.08				
**Free water clearance (ml/min/kg BW)**	**male**	20.0	27.9	3.3	NS	<.001	NS
	**female**	34.7	35.2				

A 24 h urine collection with paired blood sample enabled analysis of renal function in offspring. Osmolarity and electrolytes were measured by an osmometer (Osmomat 030, Gonotec) and ICP-MS (XSeries II, Thermo Fisher, Ltd), respectively. Data are estimated marginal means plus the standard error of the differences between means (s.e.d.) for: Control diet (−ve salt, n = 7 dams), n = 13/13 male/female offspring; 4% salt (+ve salt, n = 5 dams), n = 6/8 male/female pups. Data were analysed by mixed effects models with treatment (control *vs.* 4% salt) and sex (male *vs.* female) or their interaction as fixed effects and dam included as a random term (Genstat v14). NS, not significant.

**Table 4:** The kidneys of maternally salt-exposed offspring appear to handle sodium appropriately.

*Baseline renal function in adult offspring at 12 weeks of age*							
		± Maternal salt			*P* value		
	Sex	−ve	+ve	s.e.d.	Salt	Sex	Salt*Sex
**Food intake (g/day/kg BW)**	**male**	64.0	67.7	8.2	NS	0.005	NS
	**female**	88.1	82.1				
**Salt intake (g/day/kg BW)**	**male**	0.16	0.17	0.02	NS	0.005	NS
	**female**	0.22	0.21				
**Water intake (ml/day/kg BW)**	**male**	71.8	77.8	4.9	NS	<.001	0.02
	**female**	111	96.8				
**Urine output (ml/day/kg BW)**	**male**	29.8	28.3	2.9	NS	<.001	NS
	**female**	40.7	44.2				
**K excretion (µmoles/h/kg BW)**	**male**	236	318	58	0.05	<.001	NS
	**female**	356	483				
**Albumin excretion (g/L/h/kg BW)**	**male**	1.18	1.00	0.28	NS	NS	NS
	**female**	0.99	1.14				
**Albumin clearance (ml/min/kg BW)**	**male**	0.59	0.44	0.12	NS	NS	NS
	**female**	0.54	0.68				
**Creatinine clearance (ml/min/kg BW)**	**male**	2.55	2.43	0.58	NS	NS	NS
	**female**	1.91	2.72				
**Osmolal clearance (ml/min/kg BW)**	**male**	0.11	0.12	0.01	NS	0.03	NS
	**female**	0.12	0.15				
**Free water clearance (ml/min/kg BW)**	**male**	30.2	28.6	3.6	NS	0.003	NS
	**female**	38.6	35.6				

Food and water intake were measured over 3 days with the average intake presented. A 24 h urine collection with paired blood sample enabled analysis of renal function in offspring. Osmolarity, creatinine/albumin and electrolytes were measured by an osmometer (Osmomat 030, Gonotec), auto-analyser (RX-IMOLA, Randox) and ICP-MS (XSeries II, Thermo Fisher, Ltd), respectively. Data are estimated marginal means plus the standard error of the differences between means (s.e.d.) for: Control diet (−ve salt, n = 6 dams), n = 6/6 male/female offspring; 4% salt (+ve salt, n = 6 dams), n = 6/6 male/female pups. Data were analysed by mixed effects models with treatment (control *vs.* 4% salt) and sex (male *vs.* female) or their interaction as fixed effects and dam included as a random term (Genstat v14). NS, not significant.

**Table 5:** The kidneys of maternally salt-exposed offspring appear to handle sodium appropriately under conditions of salt-loading.

*Salt-stimulated renal function in adult offspring at 12 weeks of age*							
		± Maternal salt			*P* value		
	Sex	−ve	+ve	s.e.d.	Salt	Sex	Salt*Sex
**Food intake (mg/day/kg BW)**	**male**	62.1	58.5	3.2	NS	<.001	NS
	**female**	81.4	78.3				
**Salt intake (g/day/kg BW)**	**male**	2.48	2.34	0.12	NS	<.001	NS
	**female**	3.25	3.13				
**Water intake (ml/day/kg BW)**	**male**	136	123	12	NS	<.001	NS
	**female**	202	203				
**Urine output (ml/day/kg BW)**	**male**	91	79	10	NS	<.001	NS
	**female**	131	130				
**K excretion (µmoles/h/kg BW)**	**male**	172	150	24	NS	0.02	NS
	**female**	222	192				
**Albumin excretion (g/L/h/kg BW)**	**male**	2.52	3.50	1.12	NS	NS	NS
	**female**	2.29	1.35				
**Albumin clearance (ml/min/kg BW)**	**male**	2.22	1.35	0.50	0.04	0.07	NS
	**female**	1.30	0.78				
**Creatinine clearance (ml/min/kg BW)**	**male**	5.98	5.50	2.31	NS	NS	NS
	**female**	6.89	5.09				
**Osmolal clearance (ml/min/kg BW)**	**male**	0.24	0.21	0.04	NS	0.004	NS
	**female**	0.37	0.32				
**Free water clearance (ml/min/kg BW)**	**male**	86.8	77.9	12	NS	<.001	NS
	**female**	132	120				

Food and water intake were measured over 3 days with the average intake presented. A 24 h urine collection with paired blood sample enabled analysis of renal function in salt-loaded offspring. Osmolarity, creatinine/albumin and electrolytes were measured by an osmometer (Osmomat 030, Gonotec), auto-analyser (RX-IMOLA, Randox) and ICP-MS (XSeries II, Thermo Fisher, Ltd), respectively as described in Methods. Data are estimated marginal means plus the standard error of the differences between means (s.e.d.) for: Control diet (−ve salt, n = 6 dams), n = 6/6 male/female offspring; 4% salt (+ve salt, n = 6 dams), n = 6/6 male/female pups. Data were analysed by mixed effects models with treatment (control *vs.* 4% salt) and sex (male *vs.* female) or their interaction as fixed effects and dam included as a random term (Genstat v14). NS, not significant.

### Hypernatraemia in maternally salt-exposed offspring is likely due to a glucocorticoid-driven increase in colonic sodium-hydrogen antiporter 3

**Figure 4:**
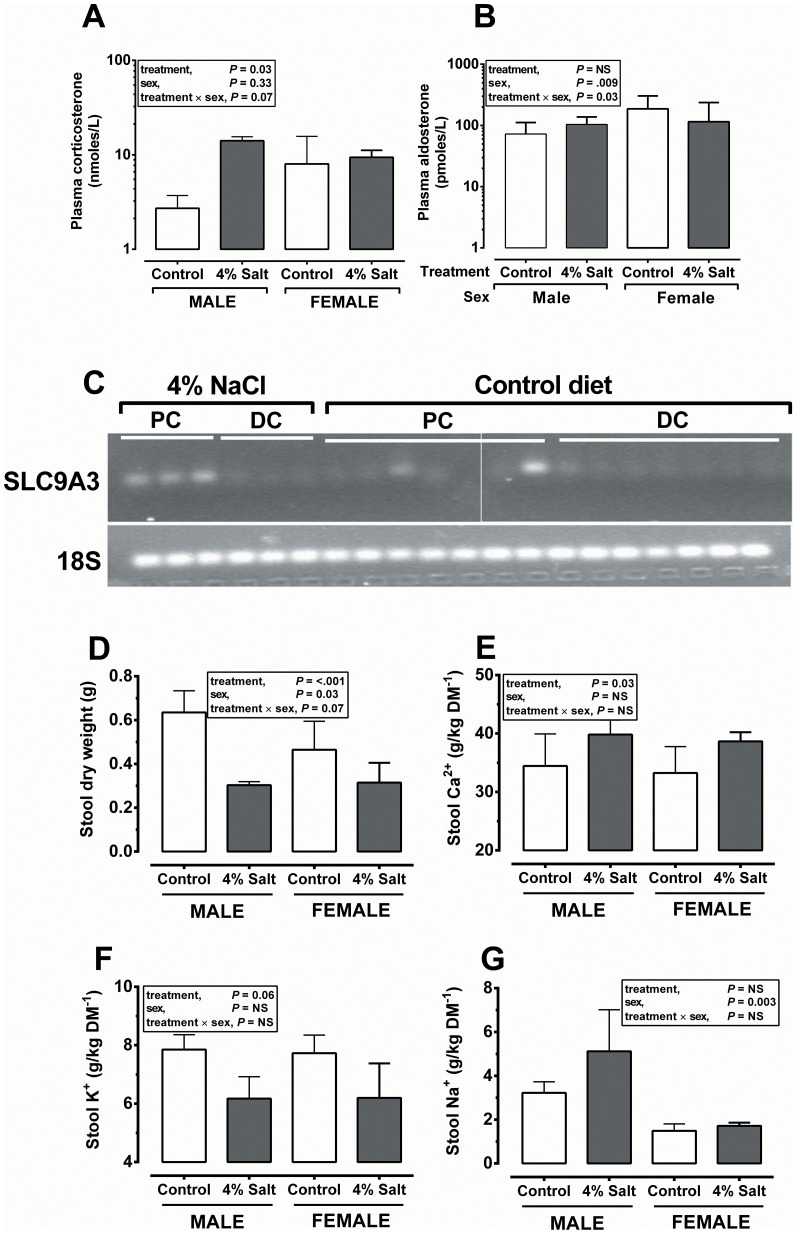
Salt-exposed offspring have increased plasma corticosterone and proximal colon SLC9A3 which influences gastrointestinal electrolyte handling. Data are presented with mean (±95% CI) indicated and are from the adult offspring of dams fed a Control (0.26% salt) diet; n = 8 dams, n = 5–7 male/female offspring or high-salt (4% salt) diet; n = 6 dams, n = 4–6 male/female offspring. Plasma corticosterone was measured by ELISA. Faecal collection and measurement of electrolytes (by ICP-MS) were as described in Methods. Data were analysed by mixed effects models with treatment (control *vs.* 4% salt) and sex (male *vs.* female) or their interaction as fixed and dam as a random effect (Genstat v14). Corticosterone was analysed as log_10_ transformed to normalise the error distribution and is shown as an antilog for clarity. NS, not significant. PC, proximal colon. DC, distal colon.

Baseline plasma corticosterone was significantly elevated (10 fold; P = 0.01) in the male offspring of prenatally salt-exposed animals (Figure 4A), with little effect on other measured steroids such as aldosterone (Figure 4B). Elevated plasma corticosterone in prenatally salt-exposed offspring was accompanied by a robust up-regulation of SLC9A3 in the proximal colon (Figure 4C) – the major mechanism for gastrointestinal (colonic) Na^+^ reabsorption (in a neutral exchange for hydrogen) which is glucocorticoid-inducible [25]. In contrast to the kidney, the distal gastrointestinal tract appeared significantly influenced by the maternal diet; we observed significantly decreased faecal wet (data not shown) and dry weight (Figure 4D; from measurement of the first individual droppings formed in the colon) with no difference in total water content (67.6±0.8 *vs.* 67.3±1.2% water) or total measured electrolytes (57.9±1.99 *vs.* 52.6±1.47 g/kg dry matter [DM] for SD vs. CD, respectively; P = NS both cases). However, analysis of individual electrolyte concentrations in faecal matter indicated subtle effects of maternal diet on offspring colonic electrolyte handling; faecal Ca^2+^ content was significantly increased (Figure 4E), there was a trend for faecal K^+^ content to be decreased (Figure 4F) and faecal Na^+^ was significantly increased in male *vs.* females, but there was no residual prenatal diet effect (Figure 4G). Faecal Mg^2+^ content was not different between high salt exposed and unexposed offspring (9.08±0.16 *vs.* 8.65±0.25 g/kg DM for SD vs. CD, respectively).

## Discussion

Moderate salt-loading of rat dams before and during pregnancy leads to hypernatraemia and marked changes to their fluid balance, but few overt effects on their offspring *in utero*. However, we show that their adult offspring, despite no direct exposure to salt diet, retain their hypernatraemic phenotype contributing toward plasma hypertonicity and hypertension – the latter effect being markedly sex-specific (males>females affected). *In vitro*, increased extracellular salt in the media bathing a developing kidney significantly impairs its growth – an effect not observed in the lung, which also grows by branching morphogenesis. *In vivo*, fetal plasma is not influenced by maternal salt diet; hence fetal kidney development under these conditions is apparently normal, resultant adult kidney function is also relatively normal with no tendency for greater salt retention to explain persistent hypernatraemia and hypertension in salt-exposed male offspring. However, our preliminary evidence suggests that maternal salt-loading at a vulnerable and transitional (neonatal) period for development of the offspring gut and brain may influence functional aspects of each to underpin hypertension as an adult. In the adult gut, maternal salt loading influenced electrolyte handling. In the adult brain we hypothesise an effect on the threshold for regulation of plasma osmolality, leading to increased plasma corticosterone (particularly in males) which further influences gastrointestinal electrolyte handling e.g. through the major glucocorticoid-sensitive sodium-coupled transporter in the proximal colon, SLC9A3.

### Programmed hypernatraemia in offspring

The level of hypernatraemia and thus increased osmolality in the offspring of salt-loaded dams despite little direct exposure is considerable, interesting and important. Increased sodium retention is commonly associated with glucocorticoid excess (apparent or otherwise) and hypertension [26]. Hence, determining a non-genetic cause of increased sodium retention is of primary importance. There are a number of potential mechanisms that could result in permanently high plasma sodium levels including 1) consistently high intake of sodium, 2) a net renal loss of water 3) relatively greater renal sodium retention or impaired excretion, or finally 4) more efficient absorption from the gastrointestinal tract. Each will be discussed in turn:

1. **Sodium intake.** In adult offspring, daily food and thus salt intake was not different between groups either under basal conditions (Table 4) or when provided with a high salt diet (Table 5) thus ruling out increased salt consumption as the mechanism for hypernatraemia in salt-exposed offspring.
2. **Increased free water clearance.** Elevated plasma osmolality is more commonly associated with diabetes insipidus (DI) in which either production of AVP is lacking (central DI) or kidneys are insensitive to AVP (nephrogenic DI) [27]; each of which increases free water clearance and sufferers of DI are characteristically polyuric and polydipsic. In our animal model, free water clearance was similar in adult offspring (Table 3), there was no evidence of polyuria or polydipsia (Table 4) and AVP levels in all groups were below the limit of detection, suggesting that the mechanisms of disease in DI are not responsible for hypernatraemia in salt-exposed offspring in our study.
3. **Increased salt-retention or decreased salt-excretion.** Plasma corticosterone was considerably elevated (approximately 10 fold) in adult offspring of salt-fed mothers (particularly males; Figure 4). High blood pressure under these conditions is thought to be partially mediated by inappropriate activation of the mineralocorticoid receptor (MR) by the higher circulating concentrations of glucocorticoid in the distal tubule, since corticosterone and aldosterone have equal affinity for MR, ultimately resulting in greater salt-retention - in *extremis* this is known as the syndrome of Apparent Mineralocorticoid Excess [26]. Such a mechanism is attractive but cannot explain our environmentally-induced phenotype; plasma aldosterone concentrations were not overtly elevated in salt-exposed offspring and, when being fed chow diet (i.e. low-salt), prenatally salt-exposed offspring had greater rates of urinary sodium and potassium excretion (but similar electrolyte and osmolal clearances) indicating an attempt by their kidneys to rectify hypernatraemia. Such effects argue against greater net salt-retention in salt-exposed offspring during consumption of chow diet. However, the challenge of salt-loading (4% NaCl diet) superimposed upon their endocrine abnormalities elicited a 10^+^-fold increase in sodium excretion in all groups but revealed a degree of impairment in the ability of salt-exposed offspring animals to clear sodium (Figure 3C), which together with similar potassium excretion at this time suggests a degree of increased renal sensitivity to aldosterone in salt-exposed offspring, perhaps facilitated by similar renal 11-beta hydroxysteroid dehydrogenase type 2 (11β-HSD2) abundance, despite the higher plasma glucocorticoid.
4. **Sodium absorption from the gastrointestinal tract.** Chronically elevated plasma sodium without any evidence of increased dietary intake or greater net renal retention suggests a more efficient continuous reabsorption at an alternative site, likely the gastrointestinal (rather than salivary) tract. It is well established that glucocorticoids regulate sodium uptake across the gut via induction of expression of a number of sodium/proton antiporters, particularly SLC9A3 at specific locations along the gastrointestinal tract including ileum and proximal colon [28]–[30]. The proximal colon is also a target for aldosterone induced transcriptional upregulation of SLC9A3 [31] and its activation in brush border membrane vesicles (BBMVs), by incubation with aldosterone, results in a considerable increase in sodium transport that is not observed when the BBMVs are derived from the ileum. Hence, glucocorticoid specific regulation of gastrointestinal SLC9A3 predominantly occurs in the ileum, whilst SLC9A3 activation in the proximal colon may be via upregulation of the MR. The chronic elevation in circulating glucocorticoid in salt-exposed offspring clearly has the potential to up-regulate SLC9A3 expression, facilitating the elevation of plasma sodium levels and blood pressure [32] in the long-term in these animals. Indeed, an increase in the expression of proximal colonic SLC9A3 in salt-exposed offspring, with negligible expression in controls, provides preliminary evidence of a potential mechanism for hypernatraemia in this group – with due acknowledgment that increased transcript expression is not always linearly followed by increased translation into greater protein abundance [33]. Furthermore, whilst we clearly observed effects on gastrointestinal electrolyte handling in the adult offspring (see Figure 4), greater sodium reabsorption i.e. less sodium in faecal matter, was not amongst them. Also, since elevated plasma osmolality itself, as well as increased glucocorticoid, can induce SLC9A3 [25], then further work beyond the scope of the current manuscript is clearly required to validate and dissect the relationship between neonatal salt exposure, gut development and gastrointestinal electrolyte handling later in life.

### Effect of maternal salt diet on structural development of the fetal kidney

Development and structure of the fetal/offspring kidney has been previously suggested to be affected by maternal high salt diet but the data are conflicting, with some groups showing a decrease in, for example, nephron number [34] while others report no change [35], [36]. In this study, we found no *in vivo* evidence to support a marked effect of maternal salt diet on offspring kidney anatomy and physiology; nephron numbers in near-term offspring (i.e. during nephrogenesis) and in adulthood (i.e. after completion of nephrogenesis) were similar between groups. We also found no evidence of renal damage in offspring kidneys, suggesting that, at least in our animals, there had been no direct structural impact on offspring kidneys of developmental exposure to a maternal high-salt diet. This variation in outcome despite apparently similar diets may reflect different experimental protocols used to measure similar endpoints or suggests that the influence of maternal salt varies with different populations (genotypes) of animals. Nevertheless, *in vitro*, it is clear that growth of cultured fetal kidneys is impaired when exposed to elevated extracellular NaCl (25 mM being approximately equivalent to the elevation observed after maternal salt-loading) in the incubating media. By 2 days, growth was significantly impaired even at the lowest NaCl dose (Fig. 2K). Furthermore, we clarified that the effect was specific to NaCL and not due to greater internal/external osmotic pressure, as incubation with 100 mM mannitol or urea, respectively failed to recapitulate the effect on kidney growth. In the absence of any differences in electrolyte composition in fetal fluid compartments, despite clear differences in maternal, argues rather strongly that the placenta has a key role protecting and modifying the delivery of charged particles to the fetus. Previous studies have shown in the late gestation rat fetus that the fetal circulation may mirror plasma sodium fluctuations in the maternal circulation in the short-term (i.e. hrs) [37]. However, after maternal administration of a NaCl bolus, recovery of fetal plasma osmolality back to baseline had occured by 9 h in the fetal but not maternal circulation, indicating an independent role for the placenta in modifying the fetal environment [37]. Indeed, our data would suggest just such an effect after long-term (days-weeks) elevated sodium exposure, given no difference in fetal electrolyte concentrations. Whilst an optimal environment for development of fetal organs may be ensured, a potential cost may be incurred to placental structure and function that may have a long-term influence on the offspring's vasculature [38].

### Effect of maternal salt diet on offspring blood pressure

Higher blood pressure after exposure to a maternal high salt diet is one of the few repeatable phenotypes within the paradigm of developmental programming [11]–[14]. However, these earlier studies often excluded a potential sex-specific effect on offspring blood pressure by studying outcome in only one sex. Disparity in maternal dietary effects on male or female offspring underpins many diverse nutritional interventions that have explored developmental programming [39]–[42]. To our knowledge, none are as marked as presented here with respect to the programming of blood pressure; we show that generation of hypertensive offspring is male-specific, females tended to be *hypotensive* – an average 25 mmHg effect size. Clearly, further investigation and experimentation is warranted, beyond the scope of the present study, to identify potential mechanisms. A testable hypothesis, however, would be to examine a potentially ‘protective and pro-survival’ action of high oestrogen levels in the female. After prenatal hypoxia, sex-specific programming of adult blood pressure (high in males but not females) was observed and the authors were able to identify an absence of oestrogen in males as the causal factor [41], [42]. For females, the protective effect was considered to have an epigenetic origin [41] and such effects may fundamentally underpin gene × environment (i.e. gender) interactions in developmental programming [43]. For example, we found that periconceptional exposure to a maternal methyl deficient diet for only 6 days (day 0–6 gestation) in sheep revealed significant sex-specific differential DNA methylation of CpG islands in the fetal livers at day 90 gestation, i.e. of the altered loci as a result of the dietary treatment, 53% were specific to male and only 12% specific to female [39].

### A mechanism involving the brain and gastrointestinal tract?

Finally, it is of interest that plasma osmolality was responsive to salt-loading in pregnant rats (i.e. it rose by 20–40 mosmoles/kg, relative to controls) but not in their offspring, despite differing baselines between groups, suggesting rigid control of osmolarity around the set-point in all groups of offspring. The implication from these data is that an upward shift in the osmotic threshold has become established in the male and female offspring of salt-loaded rats and is fully-functional at that level (i.e. a reset central osmostat) – negligible plasma vasopressin levels, despite high plasma osmolality, suggests adequate volume control at this elevated level [44]. In sheep, experimental maternal dehydration through mid-late gestation that elevated maternal osmolality from 313 to 325±3 mosmol/kg H_2_O, leaves the subsequent lambs with elevated plasma osmolality (due to hypernatraemia) – which is maintained even after infusion with hypotonic saline – and hypertension [45]. Thus two disparate experimental paradigms in different species (sheep and rat) appear to induce a similar phenotype in the adult offspring (increased plasma osmolality and blood pressure) that we show here for the first time has a marked sex-specificity; maternal hypernatraemia *leads to* offspring hypernatraemia (male and female) *and* hypertension (male only). It is important to put in a comparative context the likely timing of the micronutrient insult relative to the developmental phase in the two species; late gestation in the fetal sheep and the neonatal period in the rat are considered particularly ‘vulnerable’ periods of brain development when similar developmental processes in the brain are occurring [46], [47]. It is known that pregnancy *per se* evokes an early and marked plasma volume expansion enabled, in part, through central downward resetting of the osmotic threshold for AVP release from the posterior pituitary [48], [49] leading to net sodium and thus fluid retention. Such a mechanism, together with increased dietary salt intake, may explain hypernatraemia in the dams in our study. A similar and attractive hypothesis, that was proposed to explain salt-sensitive hypertension, may account for hypernatraemia and hypertension in the offspring in our model: if neonatal exposure to excess salt translates into excess cerebrospinal fluid sodium then exacerbated local aldosterone and angiotensinergic action in the brain may collectively alter the central osmostat, activate sympatho-excitatory afferents leading to increased plasma cortisol, high blood pressure and other sequalae [50]. This aspect of the phenotype requires clarification in further studies but offers a window into a possible mechanistic pathway for the nutritional programming of high blood pressure in laboratory animals.

## Perspective

From a nutritional perspective, one price of progress is an inevitable and inescapable dietary intake of excess salt, which increases the blood pressure of an individual consuming excess salt but also the next generation exposed *in utero*. The prevailing scientific literature suggests programming of kidney development and function primarily underpins this phenotype. We provide an alternative hypothesis: at a developmentally vulnerable period for the brain and gut in the rat i.e. during transition from parenteral to enteral feeding and the necessary physiological adaptation required in the offspring gut-brain axis, then increased salt exposure at this time – here, passively via the dam – may detrimentally affect this axis to have longer-term effects on the osmotic and pressor balance of the adult offspring. We acknowledge increased glucocorticoid action in males may, in part, underpin the sex-specificity of our phenotype but cannot ascribe cause or effect to this response since it is likely that many other endocrine pathways such as local renin-angiotensin-aldosterone action may equally be involved. Future work can begin to tease apart these multivariate effects. In conclusion, our study adds weight to the argument that salt intake should be reduced *per se* but particularly in the range of foods consumed by vulnerable babies and neonates.

## Supporting Information

Figure S1 **Increased extracellular salt has no effect on in vitro lung growth.** **A–R**: representative images of lungs (n = 4–6 replicates) cultured for 3 days in media with varying osmolality, generated using NaCl, mannitol or urea, at concentrations indicated on *y*-axes.
(JPG)

Figure S2 **Renal structure and hormone output is unaffected by maternal diet.** A,B; representative histological sections (×200, periodic acid shiff) from a single adult offspring (8-weeks of age) from dams fed a control, low-salt, (A) or high-salt (B, 4%) diet. C–F; data are for offspring of dams fed control diet (Control, n = 8 dams; n = 5–7 males/females) or 4% salt diet with water *ad libitum* (4% Salt, n = 6 dams; n = 4–6 males/females). Plasma and urinary steroid hormones were measured by a rodent specific ELISA in plasma and urine collected after 24 h in a metabolic crate. Data are presented with mean (±95% CI) indicated and were analysed by mixed effects models with treatment (control *vs.* 4% salt) and sex (male *vs.* female) or their interaction as fixed effects and dam as a random effect (Genstat v14). Steroids were analysed as log_10_ transformed to normalise the error distribution and are shown as antilogs for clarity. NS, not significant.
(TIF)
