# Supplementary figures and images for: Excess Maternal Salt Intake Produces Sex-Specific Hypertension in Offspring: Putative Roles for Kidney and Gastrointestinal Sodium Handling
Source: PLoS One. 2013 Aug 22;8(8):e72682. doi: 10.1371/journal.pone.0072682 (PMC3749995; doi:10.1371/journal.pone.0072682)

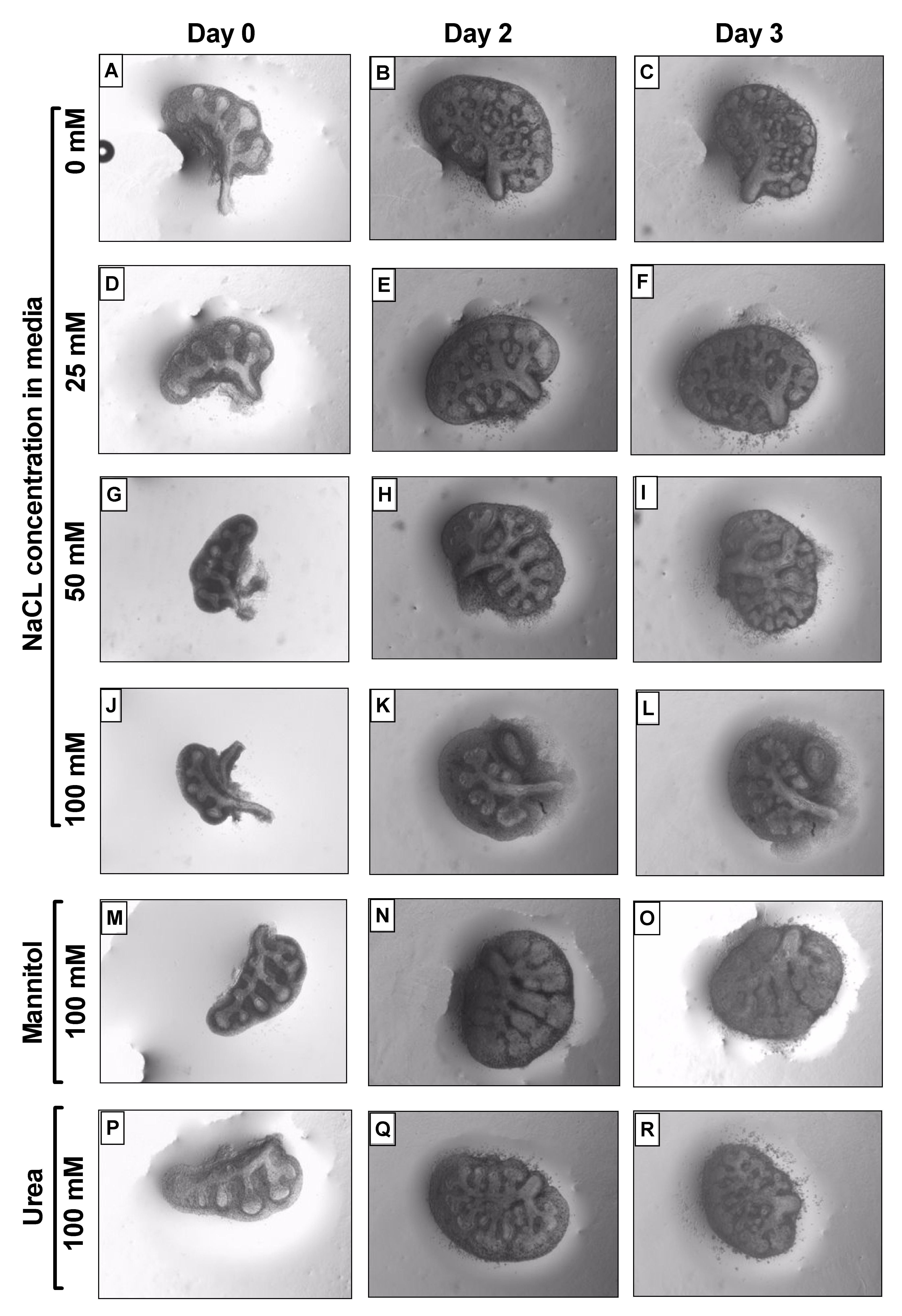

Supplement: Figure S1 — Increased extracellular salt has no effect on in vitro lung growth. A–R: representative images of lungs (n = 4–6 replicates) cultured for 3 days in media with varying osmolality, generated using NaCl, mannitol or urea, at concentrations indicated on y-axes. (JPG) [file pone.0072682.s001.jpg]
